# Ranking lambs by feed efficiency – are there changes in ruminal fermentation parameters and enteric methane emissions?

**DOI:** 10.1007/s11250-026-05257-6

**Published:** 2026-07-28

**Authors:** Charleni Crisóstomo, Danielle Nunes Gurgeira, Luiza Vage Coelho Sartorib, Claudia Cristina Paro de Paz, Josiel Ferreira, Adibe Luiz Abdalla, Ricardo Lopes Dias da Costa

**Affiliations:** 1 Laboratório de Nutrição Animal, Centro de Energia Nuclear na Agricultura (CENA), Universidade de São Paulo (USP), Piracicaba, 13400970 SP Brazil; 2 Instituto de Zootecnia, Centro de Pesquisa e Desenvolvimento de Zootecnia Diversificada, Nova Odessa, 13380011 SP Brazil; 3 Centro de Pesquisa Pecuária Sustentável, Instituto de Zootecnia, São José do Rio Preto, 15130000 SP Brazil; 4 Instituto Federal de Educação, Ciência e Tecnologia Goiano, Campus Campos Belos, Campos Belos, GO 73840000 Brazil

**Keywords:** CH_4_, Greenhouse gases, Protozoa, Residual feed intake, Santa Inês breed

## Abstract

This study investigated the influence of feed efficiency on ruminal fermentation parameters and methane (CH_4_) emissions in Santa Inês lambs. Forty intact male lambs were classified into three feed efficiency groups based on residual feed intake (RFI) and weight gain (RIG): low (RFI + and RIG-), medium (RFI ± and RIG±), and high (RFI- and RIG+). The experimental trials were conducted in two phases with controlled feeding and CH_4_ emissions measured in individual chambers. Ruminal fermentation was assessed through ruminal fluid sampling and protozoa counting. Data were analyzed using repeated measures mixed models. No significant differences (*P* > 0.05) were observed among groups for dry matter intake, body weight, and CH_4_ emissions. Numerically, high-efficiency lambs (RIG+) emitted 15.2% less CH_4_ per kg of dry matter intake, highlighting the relationship between lower emissions and higher efficiency. The lack of variation in fermentative parameters suggests that a uniform diet may have limited significant differences among groups with varying efficiency. In addition, these results indicate that RFI and RIG classifications are sensitive to the animals’ life stage and may show limited consistency across evaluation periods.

## Introduction

Improving ruminant feed efficiency significantly impacts economic performance and reduces environmental impacts by lowering feeding costs and emissions associated with livestock (Leão et al. 2021; Ferreira et al. 2024). Approximately 11% of global methane (CH_4_) emissions originate from enteric fermentation (Rasmussen and Harrison 2011). Consequently, livestock breeding contributes significantly to greenhouse gas production, primarily through enteric CH_4_ from ruminant digestion, which contributes to global warming and is a precursor to tropospheric ozone formation. Residual feed intake (RFI) was proposed by Koch et al. (1963) and has been used as a selection criterion for ruminants to increase individual feed efficiency (Ferreira et al. 2024) since efficient animals consume less feed than expected for their weight and rate of gain than do their more inefficient (or low-RFI) counterparts (Carberry et al. 2012). However, RFI does not consider the animal’s weight. For this limitation, Berry and Crowley (2012) proposed a measure of feed efficiency, residual feed intake and gain (RIG), which takes into account both the RFI and the weight gain. The RIG identify fast-growing animals, which, at the same time, consume less food than the average intake of the population, without differences for body weight.

Volatile fatty acids produced in the rumen provide a large portion of a ruminant’s energy requirements and play a crucial role in productive aspects. Studies such as Zhang et al. (2021) and Zeng et al. (2023) suggested that ruminal fermentation parameters, among other aspects, can vary according to feed efficiency. These modifications are associated with an abundant and diverse microbiome, influencing greenhouse gas emissions and the production of volatile fatty acids. Despite the interesting results mentioned above, studies on feed efficiency for these research purposes remain scarce, as efforts are typically focused directly on the productive aspects of ruminants.

Given these expectations, this study aimed to evaluate ruminal fermentation parameters and enteric CH₄ emissions in hair sheep classified according to feed efficiency estimates. The hypothesis tested was that efficient sheep exhibit lower dry matter intake (DMI) and CH_4_ emissions, while maintaining similar average daily gain (ADG) compared to less efficient sheep, potentially due to metabolic processes associated with altered ruminal fermentation parameters.

## Materials and methods

### Local of the experiment

The first experimental trial was carried out in a sheep confinement area located in the Sheep Sector of the *Instituto de Zootecnia* of the Paulista Agribusiness Technology Agency, belonging to the Secretariat of Agriculture and Supply, located in the municipality of Nova Odessa, São Paulo State (22º46’39’’S and 47º17’45’’W and altitude of 570 m) and the second experimental trial in the Animal Nutrition Laboratory (LANA) of the Center for Nuclear Energy in Agriculture (CENA) from the University of São Paulo (USP), located in the city of Piracicaba, SP (22°23’31’’S, 47°38’57’’W).

### Animals, feeding, management and feed efficiency classification

For the first experimental trial, forty uncastrated male Santa Inês lambs, with a mean initial age of 120 days and an average initial body weight of 30.4 ± 2.46 kg, were used. The animals were allocated to a collective pen, where they remained for a 15-day adaptation period, consuming the experimental diet for lambs using in all experimental trial (Coopermota, Cândido Mota, SP), composed of 90% concentrate mixture (corn grain and soybean meal) and 10% Tifton-85 hay (*Cynodon* spp.) (Table 1). The facility contained nine automatic feeders and two automatic drinkers, both connected to an electronic animal weighing scale. The animals were weighed every time they ate or drank water (Intergado^®^, Contagem, MG, Brazil), using radio frequency identification (RFID) technology. The feed efficiency trial was conducted over a total period of 136 days, divided into two stages. At the end of the first stage (Trial 1: 0–59 days), the lambs weighed 43.5 ± 3.70 kg, and at the end of the second stage (Trial 2: 61–136 days), they weighed 58.2 ± 4.16 kg. Both stages fulfill the required duration for assessments, as outlined by Amarilho-Silveira et al. (2022) and Zeng et al. (2023).

**Table 1:** Bromatological composition of the total diet (90% concentrate mixture, 10% Tifton-85 hay) provided to the animals during the experimental period. Values expressed in g/kg of dry matter (DM)

Parameter (%)	Content
Dry matter	88.6
Crude protein (%)	20.3
Crude fiber	9.33
Ether extract	3.2
Mineral matter	6.4
Acid detergent fiber	11.7
Neutral detergent fiber	20.3
Cellulose	9.5
Hemicellulose	8.6
Lignin	2.2
Total digestible nutrients	76.3

At the end of each experimental trial, the animals were classified into three groups: RFI- (high feed efficiency), RFI± (medium feed efficiency), and RFI+ (low feed efficiency). This classification was based on the differences between observed and predicted values of DMI, calculated using ADG and metabolic body weight (BW⁰·⁷⁵), as described by Koch et al. (1963): Predicted DMI = β₀ + β₁ × ADG + β₂ × BW^0.75^, where, β₀ is the regression intercept, β₁ is the partial regression coefficient for ADG, and β₂ is the partial regression coefficient for BW^0.75^. In addition, the animals were also classified according to RIG: RIG+ (high feed efficiency), RIG± (medium feed efficiency), and RIG- (low feed efficiency). The calculation was performed based on the method described by Berry and Crowley (2012). The residuals of RFI and RIG were standardized beforehand (mean = 0 and standard deviation = 1) using the STANDARD routine (SAS, 2012). Lambs were classified as high-RFI and high-RIG (> 0.5 SD above the mean), medium-RFI and medium-RIG (± 0.5 SD of the mean) and low-RFI and low-RIG (< 0.5 SD below the mean).

### Methane emission measurement

For the second experimental trial, CH_4_ emissions were measured in the same animals in vivo using open-circuit respiratory chambers developed by LANA-CENA/USP. The assessment of gas emissions was performed over three days, with one day for adaptation and two for collection. These chambers are based on a low-cost, locally available system adapted from conventional metabolism cages for sheep, as described by Abdalla et al. (2008). Each individual chamber measured 157 × 71 × 167 cm (1.9 m^3^) and was sealed on the sides and top using 0.3 mm polyethylene sheets, leaving only the bottom open. A front inlet (5 cm diameter) allowed fresh air to enter, while an exhaust pump connected to a rear outlet (also 5 cm diameter) continuously removed air at a flow rate of 168 L/min. A peristaltic pump collected outlet air at 100 mL/min into 5-L aluminized sampling balloons. Air circulation within the chamber was maintained by a small fan, and temperature and relative humidity were monitored regularly to ensure animal comfort. The CH_4_ concentration in the sampled air was analyzed using a gas chromatograph (Shimadzu GC-2014). This setup allows for reliable and repeatable quantification of CH^4^ emissions while maintaining environmental conditions comparable to those in the rumen.

The sheep were offered the same diet used in the feed efficiency evaluation trial, divided into two meals (morning and afternoon), with ad libitum access to water. Feed refusals were weighed daily, and intake was adjusted to maintain approximately 10% orts. After 60 days, average dry matter intake was 1.25 ± 0.307 kg/day, increasing to 1.43 ± 0.372 kg/day by day 136.

### Ruminal fluid collection and analysis

At the end of each gas collection period, ruminal fluid was sampled from animals four hours postprandial using an esophageal tube. The fluid (60 mL) was stored at -20 °C for later analysis of short-chain fatty acids (SCFA) and ammonia-nitrogen (NH₃-N). Protozoa counts were performed using a diluted ruminal fluid sample stained with methyl green and analyzed under an optical microscope in a Neubauer chamber, following Dehority et al. (1983). SCFA concentrations were quantified using gas chromatography (Shimadzu GC-2010) based on Palmquist and Conrad (1971) with adaptations from Lima et al. (2018). NH₃-N levels were determined using the micro-Kjeldahl method with steam distillation (Preston 1995). Results, expressed as parts per million (ppm) and percentages, include SCFA composition, acetate ratio, and individual fatty acids such as acetic, propionic, butyric, and valeric acids.

### Statistical analysis

Statistical analyses were performed using SAS^®^ software (version 9.4; SAS Institute Inc., Cary, NC, USA). Prior to analysis, data were evaluated for normality and homogeneity of variances using the Shapiro–Wilk and Levene’s tests, respectively. As assumptions were met, no data transformation was required.

The study was considered an observational study based on post hoc classification of animals according to feed efficiency indexes. Animals were classified into feed efficiency groups separately according to residual feed intake (RFI: low, medium, and high efficiency) and residual intake and gain (RIG: low, medium, and high efficiency).

Performance variables, ruminal fermentation parameters, protozoa counts, and methane emissions were analyzed using repeated measures mixed models (PROC MIXED, SAS). Separate statistical models were fitted for RFI and RIG classifications to avoid collinearity between feed efficiency indexes, since RIG is partially derived from RFI.

For each analysis, the model included the fixed effects of feed efficiency group, and trial period. Animal was included as a random effect, with repeated measurements over time specified within animal. Repeated measures were modeled using trial period as the repeated factor within animal. The statistical model was: Y_ijkl_ = µ + G_i_ + T_j_ + Ak_(i)_ + ε_ijl_, where Y_ijkl_ = dependent variable; µ = overall mean; G_i_ = fixed effect of feed efficiency group (RFI or RIG classification); T_j_ = fixed effect of trial period; Ak_(i)_ = random effect of animal within efficiency group; and ε_ijl_ = residual error.

Different covariance structures [compound symmetry (CS), autoregressive first order (AR(1)), and unstructured (UN)] were tested, and the best-fitting structure was selected according to the Akaike Information Criterion corrected for small sample size (AICc). Least square means were compared using Tukey-adjusted tests.

Pearson correlation coefficients among methane emissions, dry matter intake, RFI, and RIG were calculated separately within each trial period using R software (R Core Team, 2024). To reduce the likelihood of spurious associations, p-values were adjusted for multiple comparisons. Correlation matrices were visualized using the ‘corrplot’ package (Wei and Simko 2024). Statistical significance was declared at *P* ≤ 0.05.

## Results

**Fig. 1:**
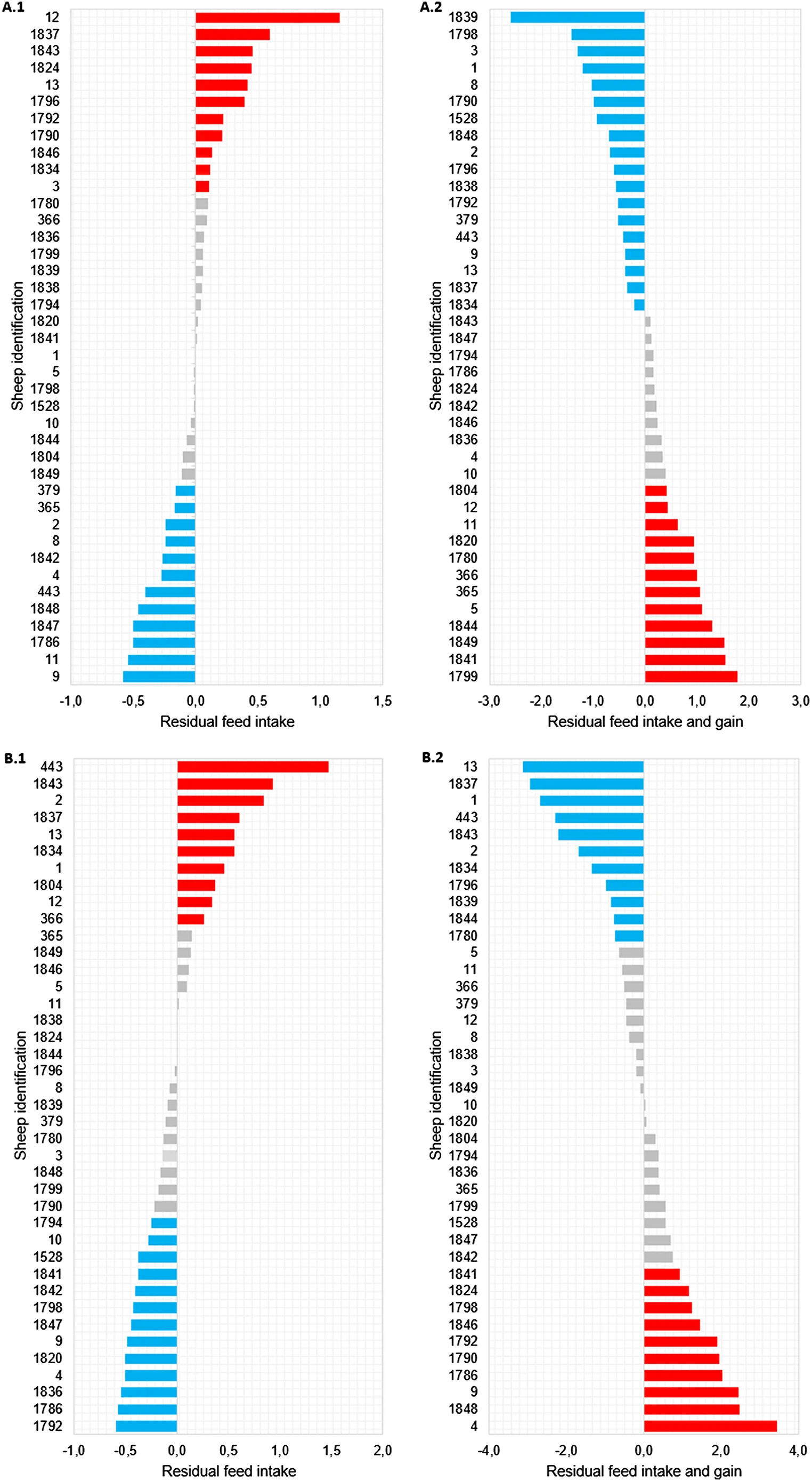
The selection of the high, medium and low residual feed intake (RFI) and residual feed intake and gain (RIG) in first (**A**) and second experimental trial (**B**)

The individual classifications in the two experimental trials, based on feed efficiency estimates, are presented in Fig. 1. In the first trial, 27.5% lambs were classified as low-RFI and 30% as high-RFI, with a larger number classified as medium-RFI (Fig. 1A.1). According to the RIG classification, more lambs were classified as efficient (Fig. 1A.2). In the second trial, there was a more balanced distribution among all RFI (Fig. 1B.1) and RIG classes (Fig. 1B.2).

The percentage of lambs that remained in the same RFI and RIG classifications between trials was low: 12.5% and 2.5%, respectively. For the least efficient animals (high-RFI and low-RIG), concordance was slightly higher for low-RIG lambs (17.5%). For medium classifications, 17.5% of lambs remained in RFI ± and 12.5% in RIG±.

Body weight (BW) and dry matter intake (DMI) differed significantly across trial periods (*P* < 0.0001), reflecting the expected increase associated with animal growth. However, no significant effects of residual feed intake (RFI) or residual intake and gain (RIG) classification were observed for BW, DMI, or methane (CH_4_) emissions (*P*> 0.05) (Table 2 and Table 3). Numerically, animals classified as RIG+ produced 15.2% less CH_4_ per kilogram of DMI per day than RIG− lambs (Table 3).

**Table 2:** Least square means of dry matter intake (DMI), body weight (BW), and methane production (CH_4_) according to residual feed intake (RFI) classification

Variable	RFI−	RFI±	RFI+	*P*-value	
				Group	Trial
DMI, kg/day	1.30	1.30	1.30	0.997	< 0.0001
BW, kg	49.7	51.0	53.5	0.336	< 0.0001
CH_4_, kg/day/BW^0.75^	0.25	0.26	0.26	0.929	NA
CH_4_, kg/day/DMI	3.5	4.0	3.9	0.761	NA

*BW*^*0.75*^ *metabolic body weight; NA not applicable*

*RFI classification: RFI- (high feed efficiency)*,* RFI± (medium feed efficiency)*,* and RFI+ (low feed efficiency)*

*Data were analyzed using repeated measures mixed models (PROC MIXED*,* SAS 9.4)*,* including the fixed effects of RFI classification and trial period*,* with animal included as a random effect*

**Table 3:** Least square means of dry matter intake (DMI), body weight (BW), and methane production (CH_4_) according to residual feed intake and gain (RIG) classification

Variable	RIG+	RIG±	RIG−	*P*-value	
				Group	Trial
DMI, kg/day	1.4	1.4	1.2	0.261	< 0.0001
BW, kg	52.4	51.3	50.5	0.729	< 0.0001
CH_4_, kg/day/BW^0.75^	0.25	0.25	0.27	0.871	NA
CH_4_, kg/day/DMI	3.5	3.6	4.2	0.544	NA

*BW*^*0.75*^ *metabolic body weight; NA not applicable*

*RIG classification: RIG+ (high feed efficiency)*,* RIG± (medium feed efficiency)*,* and RIG- (low feed efficiency)*

*Data were analyzed using repeated measures mixed models (PROC MIXED*,* SAS 9.4)*,* including the fixed effects of RIG classification and trial period*,* with animal included as a random effect*

**Fig. 2:**
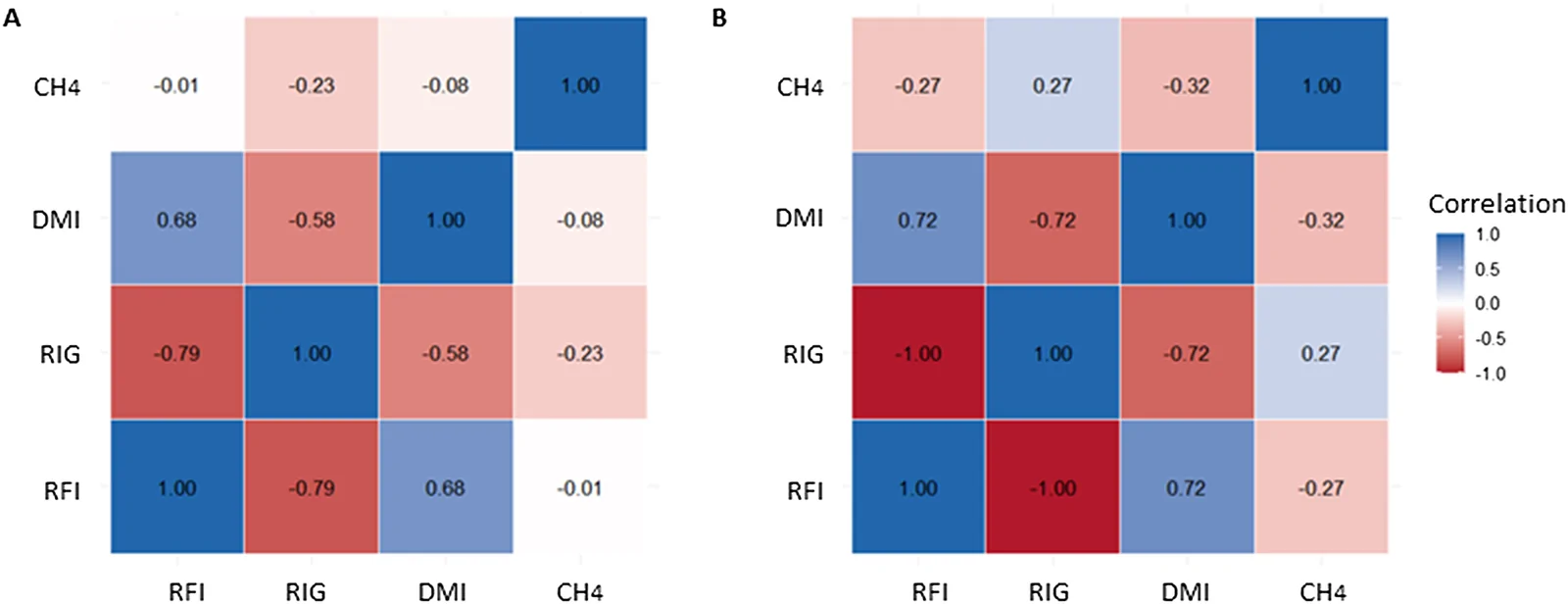
Pearson correlation coefficients between residual feed intake (RFI), residual intake and gain (RIG), dry matter intake (DMI), and enteric methane production (CH_4_) of sheep in the first (**A**) and second (**B**) experimental trials. Correlations were calculated separately within each trial period. Significant correlations (*P* ≤ 0.05, after adjustment for multiple comparisons) are indicated, while non-significant correlations are marked accordingly

Correlation coefficients ranged from low to high depending on the variables evaluated (Fig. 2).

**Fig. 3:**
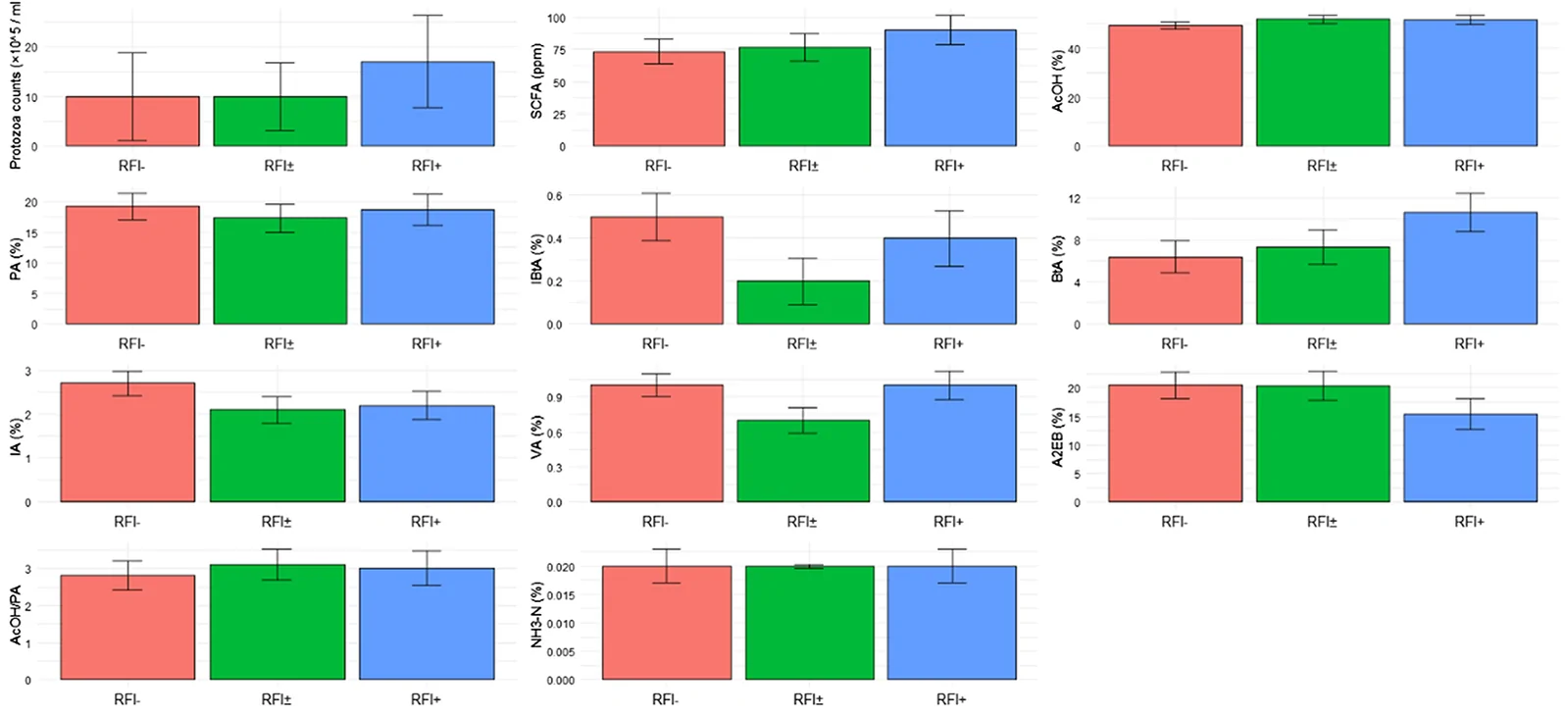
Least square means ± standard error of protozoa counts and ruminal fermentation parameters of sheep according to residual feed intake (RFI) classification: low-RFI (high efficiency), medium-RFI, and high-RFI (low efficiency). PA – propionic acid; IA – isovaleric acid; AcOH/PA – acetic/propionic acid ratio; SCFA – short-chain fatty acids; IBtA – isobutyric acid; VA – valvaleric acid; NH_3_-N – ammoniacal nitrogen; AcOH – acetic acid; BtA – butyric acid; A_2_EB – 2-ethylbutyric acid

**Fig. 4:**
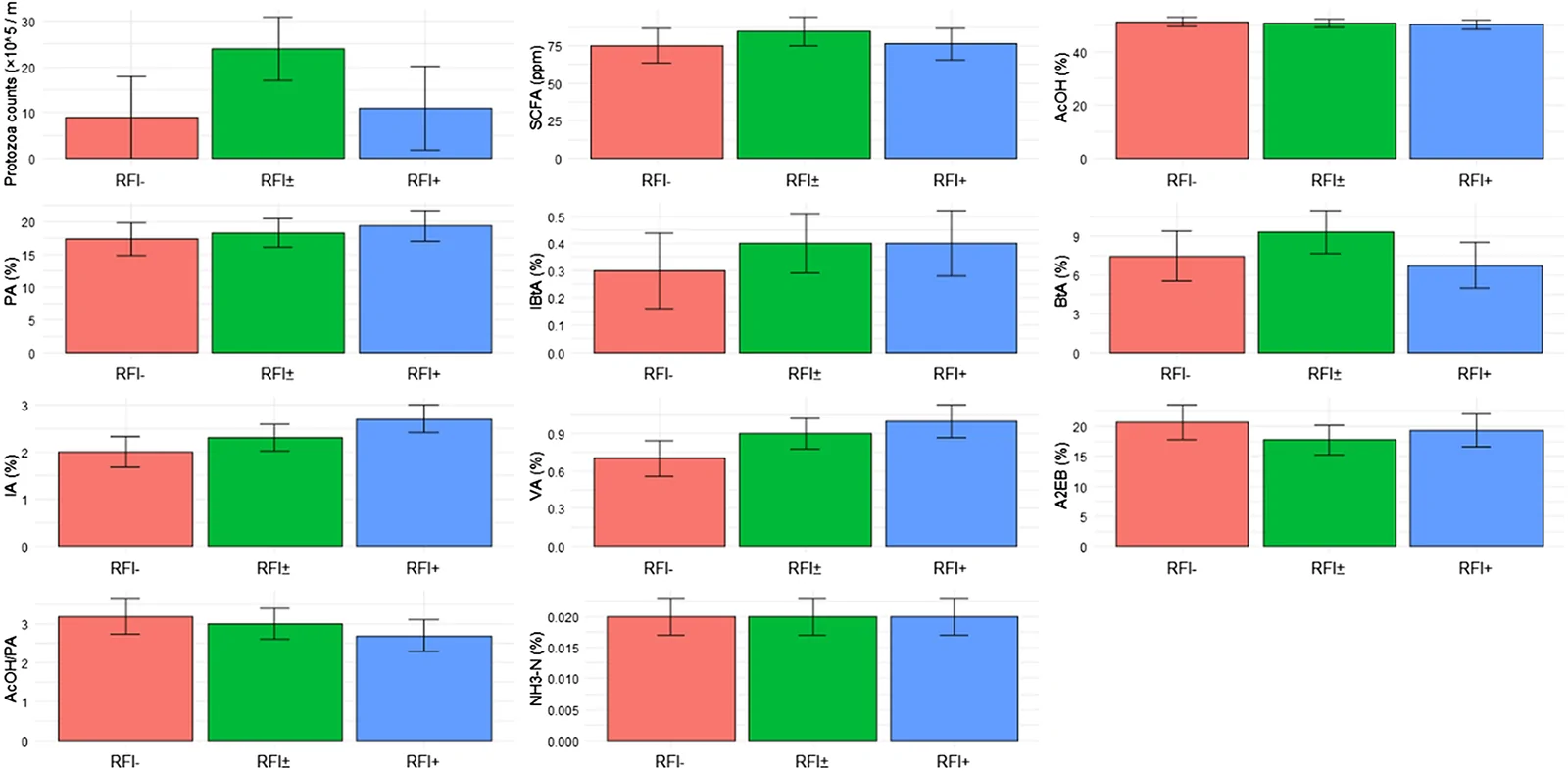
Least square means ± standard error of protozoa counts and ruminal fermentation parameters of sheep according to residual intake and gain (RIG) classification: high-RIG (high efficiency), medium-RIG, and low-RIG (low efficiency). PA – propionic acid; IA – isovaleric acid; AcOH/PA – acetic/propionic acid ratio; SCFA – short-chain fatty acids; IBtA – isobutyric acid; VA – valvaleric acid; NH_3_-N – ammoniacal nitrogen; AcOH – acetic acid; BtA – butyric acid; A_2_EB – 2-ethylbutyric acid

Protozoa count and ruminal fermentation parameters (e.g., short-chain fatty acids, NH₃-N) did not differ (*P* > 0.05) among lambs classified by RFI or RIG (Figs. 3 and 4).

## Discussion

The present study provides insights into the relationships among feed efficiency, ruminal fermentation, and CH_4_ emissions in Santa Inês lambs. Although no significant differences were detected for most parameters across RFI and RIG groups, important tendencies were observed and merit discussion.

The low consistency in feed efficiency classification (RFI and RIG) across trials demonstrates the influence of age and physiological stage on efficiency metrics. Similar findings have been reported by Amarilho-Silveira et al. (2022), emphasizing that feed efficiency estimates may not be stable over time. This raises concerns regarding the optimal timing for evaluating animals, especially in breeding programs aiming to incorporate efficiency traits.

Notably, while absolute CH_4_ emissions did not differ among efficiency classes, the most efficient lambs RIG+ produced 15.2% less CH_4_ intensity. This aligns with reports suggesting that CH_4_ intensity (emissions per unit of intake) may be a more sensitive measure of environmental efficiency than gross emissions (Zeng et al. 2023). The absence of statistical significance may be associated with high inter-individual variability and the limited sample size—a common limitation in trials aiming to detect metabolic differences.

The lack of significant differences in CH_4_ emissions among feed efficiency groups can likely be attributed to the use of a uniform, high-concentrate diet. Diets rich in readily fermentable carbohydrates promote a shift in ruminal fermentation toward propionate production, which acts as a hydrogen sink and reduces the availability of hydrogen for methanogenesis. Consequently, CH_4_ production tends to decrease and become less variable across animals. This metabolic environment may act as a “ceiling effect,” homogenizing ruminal fermentation patterns by limiting variation in hydrogen production, protozoal activity, and substrate utilization. Under such conditions, potential biological differences among feed efficiency groups may be masked, making it difficult to detect treatment effects on CH_4_ emissions. In contrast, forage-based diets typically promote greater acetate production and hydrogen release, increasing the potential for variation in methanogenesis. Therefore, differences in CH_4_ emissions associated with feed efficiency may be more detectable under diets with higher forage inclusion. Additionally, the short adaptation period to the respiration chambers (one day) may have influenced the stabilization of feed intake, stress responses, and gas exchange. Future studies should therefore consider longer adaptation periods and evaluate diets formulated to modulate protozoal populations, given their central role in hydrogen transfer and methanogenesis.

Beyond the effects of diet and adaptation, the observed variability underscores the complex biological nature of feed efficiency. Enteric CH_4_ production is governed not only by feed intake and digestion efficiency but also by the structure and activity of ruminal microbiota, nutrient absorption, and host metabolism. Although our study did not include microbial community analysis, previous research indicates that more efficient ruminants may harbor distinct microbial populations, with reduced abundance of hydrogen-producing protozoa and methanogenic archaea (Carberry et al. 2012; Zhang et al. 2021). This microbial diversity affects fermentation end-products and energy losses through CH_4_. Therefore, future research should combine metagenomic, transcriptomic, and metabolomic approaches to better understand the microbiological and biochemical mechanisms that underpin variation in feed efficiency and CH_4_ production.

The absence of differences in volatile fatty acid profiles or protozoa counts further supports the hypothesis that the rumen environment was relatively homogeneous under the given dietary conditions. Protozoa, which are key hydrogen producers, are closely associated with methanogenesis, and their abundance can strongly influence CH_4_ formation. However, the stable protozoa populations across efficiency groups in our study suggest that the diet composition and feeding management may have limited the natural variability in microbial community structure.

From a practical standpoint, these results highlight the need to refine methodologies for evaluating feed efficiency in small ruminants. The low repeatability of efficiency classification between trials (12.5% for RFI and 2.5% for RIG) indicates that external factors—such as diet composition, animal growth phase, and environmental conditions—can significantly affect the consistency of these metrics. Therefore, future selection programs should aim to standardize the timing, duration, and conditions of efficiency evaluations to improve classification accuracy and genetic parameter estimation. Moreover, the inclusion of CH_4_ intensity (CH_4_ per unit of DMI or ADG) as a complementary trait in breeding programs may provide valuable insight into environmental efficiency without compromising productivity.

Although the discussion extends the interpretation to cattle, it is essential to recognize that sheep and cattle differ markedly in digestive physiology and production systems. Cattle typically exhibit higher feed intake, longer digesta retention time, and greater fiber fermentation capacity, resulting in higher absolute CH_4_ emissions. However, the fundamental biological principles remain similar, and sheep represent a valuable experimental model for understanding ruminal efficiency and emission dynamics under controlled conditions. Translating findings from sheep to cattle must consider species-specific differences in feed intake regulation, metabolic rate, and microbial ecology, which can alter both efficiency and emission responses.

Finally, these results also highlight the importance of integrating efficiency traits with broader sustainability indicators. Genetic selection for feed efficiency can reduce production costs and CH_4_ emissions, but overemphasis on single-trait selection may inadvertently affect other economically important traits such as fertility, adaptability, or resilience. Balanced breeding objectives that consider both productive performance and environmental outcomes will be essential to ensure long-term sustainability of ruminant production systems.

## Conclusions

In conclusion, the results of this study indicate that classifications based on RFI and RIG did not significantly affect CH_4_ emissions, protozoa counts, or ruminal fermentation parameters in lambs evaluated across two sequential feed efficiency tests. One possible explanation for the absence of differences in CH_4_ production is the uniformity of the diet offered, which may have minimized individual variability in fermentative patterns. Additionally, the relatively small sample size and the short adaptation period to the respiration chambers may have contributed to the lack of statistical significance.

Although these findings are specific to lambs, caution must be taken when extrapolating them to cattle, given the physiological and digestive differences between species. In cattle, where feed intake and ruminal fermentation dynamics differ, the relationship between feed efficiency and CH_4_ emissions may follow distinct patterns. Although the sample size was limited, the controlled environment, uniform diet, and repeated-measures design strengthen the reliability of the observed trends.

From a practical standpoint, our results reinforce the complexity of identifying consistent biological markers of feed efficiency across different stages of growth. Nevertheless, the use of RIG, which incorporates both intake and weight gain, may offer a more stable and practical alternative to RFI in selecting efficient animals with potentially lower CH_4_ emission intensity, contributing to more sustainable ruminant production systems. These findings support the integration of RIG into selection protocols, particularly in intensive systems seeking to reduce CH_4_ emissions without compromising animal performance.

Some of the limitations of this study—particularly the small sample size from a genetic improvement perspective—are recognized. However, our primary objective was to generate preliminary evidence to support the identification of physiological biomarkers of feed efficiency. These insights may inform the design of future research with larger populations and longer evaluation periods.

Moreover, future studies should consider dietary strategies, chamber adaptation time, and physiological stage as experimental design factors that may influence the consistency of efficiency estimates. Integrating these aspects with genetic, microbiological, and metabolic analyses will be crucial to advancing the understanding of the biological mechanisms linking feed efficiency and CH_4_ emissions in small ruminants. Such integrative approaches will also support the development of selection programs that combine productivity and environmental sustainability, thereby enhancing the efficiency and resilience of future ruminant production systems.

## Data Availability

The authors have chosen to make it available upon request.
